# Isochrones as Indicators of the Influence of Traffic in Public Health: A Visual Simulation Application in Ávila, Spain

**DOI:** 10.3390/ijerph121012556

**Published:** 2015-10-09

**Authors:** F. Javier Otamendi, David García-Heredia

**Affiliations:** Universidad Rey Juan Carlos, Paseo Artilleros s/n, 28032 Madrid, Spain; E-Mail: d.garciahe@alumnos.urjc.es

**Keywords:** isochrones, golden hour, ambulance deployment, small towns

## Abstract

It is well known that excessive rescue times after traffic accidents negatively affect the health of those injured. There is a need to quantitatively measure the impact of unexpected events like ambulance availability, weather, floating population and congestion in those rescue times. A family of indicators based on isochrones is disguised and proposed to understand the risk of the whole population as the probability of not being assisted on time. Indicators of health risk for local towns are also defined. The indicators are calculated using a simulation model and visualized in web format. The framework of analysis is validated using Ávila (Spain) and the problem of the optimal deployment of ambulances as a test-bench.

## 1. Introduction

Traffic may affect public health in many different ways. From pollution [1] to driving behavior [2], the studies are varied. Another dimension is that of traffic accidents and their impacts in daily lives, like stress based on traffic congestions [3], or the economic burdens on the society (medical bills, disabilities, loss of lives…) [4]. We are concerned in this case with the necessity to reduce the negative impacts on the well-being of the inhabitants due to an excessive assistance time of the emergency units of different types to the people involved in an accident. This response time should be as short as possible since it is well known that “the quicker the rescue, the higher the possibility of the patient recovering”. The Golden Hour principle is widely accepted as a requisite for public health, that is, the probability of recovering greatly decreases if a traffic accident is not properly assisted within one hour [5]. Moreover, the importance of a quick medical response has been demonstrated in several studies, including a suggestion that a 10 minute reduction will greatly reduce the number of fatalities [6].

Many factors may affect this reaction time, all of which are dynamic and probabilistic in nature. Weather conditions may slow travelling times, for example, snow [7] and fog [8]. Road conditions and layout, especially on rural areas [9], or even congestions [10] may force the drivers to reduce speed, stop or take a different longer route. On top of that, spectacles and events, or holiday periods increase the population in the surroundings, as well as the congestions, raising the probability of accidents.

Accidents are therefore unexpected in nature and require for a quick assistance time of an available emergency unit with its corresponding team. If several accidents occur simultaneously, the response time is likely to increase since there will not usually be enough units to assist all the accidents at once. The movement of the ambulances is the key of the emergency system since it is very important to note that the ambulance that leaves the hub to assist an accident is not available to give service to any other accident until reaching the base again. In other words, any ambulance is unavailable during a period of time. In the case of a given region staffed with just one ambulance, the whole population is at risk whenever that lonely ambulance is not at the base. This reasoning is critical for understanding the rescue system and for the development of the framework of analysis of emergency systems, which should be comprised of a modelling tool and a set of reliable indicators.

Therefore, the focus of this article is to provide a framework for the definition and calculation of a set of indicators that are useful to measure the impact of traffic accidents in public health. Indeed, the population that is at risk is the one that could not be covered by any ambulance within the golden-hour rule, or a 60-minute threshold. Several other values of that specific time threshold, probably tighter, could however be stated, leading to the definition of a “population at risk” indicator as “the percentage of the population that lives outside a given time threshold from the hub of resources” [11]. In Spain, for example, and according to the Spanish authorities’ objectives, the population coverage should be 80% within 15 minutes [11]. In France, the Val-de-Marne department seeks a “time to rescue” of 20 minutes [12].

Within this paradigm of “population at risk as a function of the rescue time”, there is a need to develop tools and quantify indicators that may be used to establish proper rescue protocols, including the staffing of ambulances. If the system under study is usually referred to as Emergency Medical System (EMS), the staffing decision is usually related as the deployment problem [13]. In other words, the number of ambulances assigned to an EMS hub has to be enough to guarantee that those involved in an accident receive attention quickly, improving the overall coverage of the population as well as its related health, but not so many units so as to incur in excessive costs.

**Figure 1:**
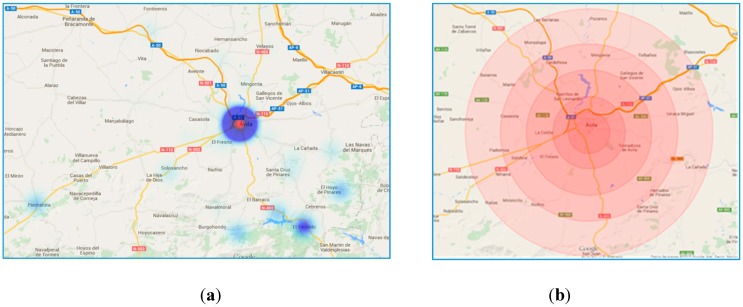
(**a**) Population dispersion: Map of roads and location of the population in the province of Ávila; (**b**) Radial isochrones.

In this context, there is a consensus following international standards that there should be 1 unit per 50000 inhabitants [11]. For example, the EMS of Ávila, the capital city of a small region that is located 100 kilometers away from Madrid and that is going to be used as a test case in this article, has one unit [14], even though the population is somewhat higher than 50,000. The region covered by the EMS hub located at the capital city of Ávila includes five main towns with over 1000 inhabitants that need to be covered within the current infrastructure of roads (Figure 1a). The blue circles are where the cities are located and its size proportional to the population. The large region causes the ambulance to cover long distances and therefore long periods of ambulances unavailability and high population risks.

**Figure 2:**
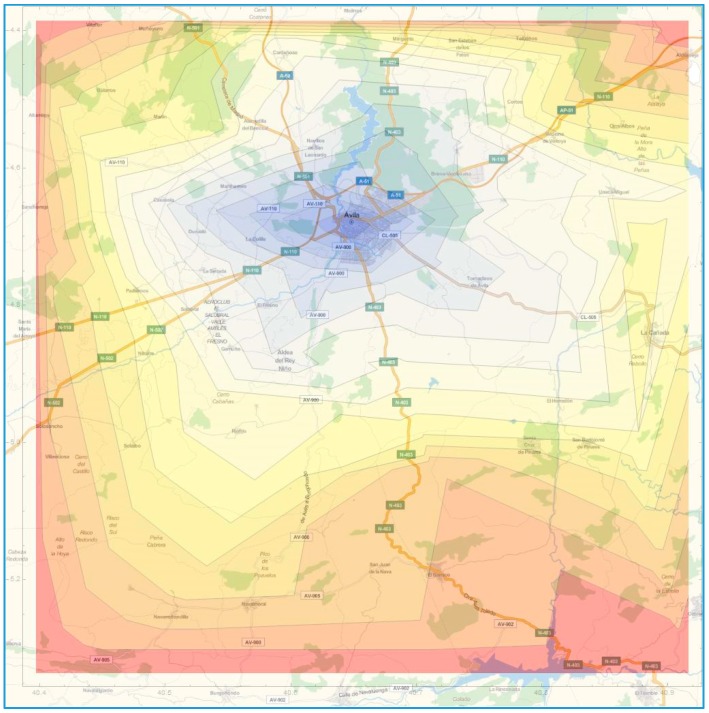
Realistic time lines.

Figure 1b includes the rescue times from the hub, assuming there is always an available ambulance at the base. The time lines are drawn as a radius following standard procedures [14]. The radius has been calculated arbitrarily for Ávila using one of the main roads under perfect traffic conditions, with GoogleMaps being the source for calculating the travel times. It looks obvious that the time distances to a given radius are not equal for each and every point, nor they are constant over time due to the different factors that have been already mentioned, like population variability and adverse driving conditions. Figure 2 shows an updated visual display with crooked time lines over the map, what gives a more realistic representation of the time reach from the hub. The color coding helps detect those areas that are under risk, with the red color indicating distances above the 60-minutes mark.

The focus of this article is therefore on the development of a new generation of time indicators that may be used to better address the reaction time problem and reduce accordingly the population at risk as a proxy of public health. These indicators include not only static conditions, but also dynamic events that negatively affect the rescue time and its corresponding representation with time lines over a web map.

From a prevention point of view, our originality is that we want to develop and define an indicator of public health that anticipate potential problems, a new indicator that relates to the proactive coverage of the population. With the proposed set of indicators the potential population under risk under dynamic conditions is better addressed especially for long- and medium-term planning.

Therefore, the article covers the definition of a family of indicators that help analyze the influence of traffic accidents in public health in Section 3. The research also includes their quantification using a simulation model, Section 4, tool that is very appropriate whenever time and unexpected events are key ingredients of the analysis. Section 5 is then used to validate the new paradigm and framework composed of a simulation model and risk indicators by using Ávila and the deployment problem, or the proper staffing of ambulances, as a test-bench. But first, let us fully define the Emergency Medical System and how it has been analyzed in the past.

## 2. Background in EMS Methods and Applications

This system of assisting traffic accidents is known as Emergency Medical System (EMS). The system works as follows. The ambulances are located at the base or hub. If an accident occurs, the ambulance is occupied (not available for another call) during a certain time period which covers the following tasks:

1. Preparation of the assistance: before leaving the base, the staff needs to prepare matters.
2. Travelling to accident location: the ambulance moves towards the scene of the accident.
3. At the accident site: the staff actuates to assist and pick the injured up.
4. Travelling to hospital: the ambulance moves from the scene towards a medical centre.
5. At the hospital: the staff actuates to drop the injured off.
6. Travelling back towards the base to report and be ready for a new call.

A thorough survey of applications of simulation to EMS was published in 2013 [15]. It divides the types of decisions into long-term (potential bases location, dimensioning of resources), mid-term (deployment problem, shift scheduling) and short term (resource dispatching, destination hospital selection, redeployment problems). A simulation optimization framework may become necessary to address the corresponding optimization problem [16] in any of the above situations.

Besides simulation, also static lineal programming models are used. A comparative study of 5 maximal accident covering models is based on data from Alberta, Canada [17]. Another review of methods relates also to the same optimization methods [18]. These models may however become inadequate when ambulances become busy [19]. In this latter article, two new models for the redeployment of ambulances are presented: the Maximal Expected Coverage Relocation Problem and the Generalized Ambulance Assignment Problem.

In terms of indicators, two types have been used in the past to quantify the level of service in EMS deployment [15]: time/distance (average response time, coverage within a standard time T, coverage within a time greater than T, round trip time, service time, vehicle utilization rate, number of calls served per vehicle/base, dispatching time, travel time to scene, waiting time, size of queue, loss ratio, overtime, total mileage) and survival cost (survival rate, cost effectiveness).

The most common one is the response time (for example, [12]), which is usually calculated as the average time that the resources take to arrive to the scene of the accident. This indicator is related to the proportion of time the response time is within the Recommended Safety Time Threshold [20]. This second indicator sometimes is called “Maximal Expected Coverage”, the measure that is complementary to the risk [21].

In terms of survival, or at least potential survival, we can mention the population risk, which is frequently stated as “the percentage of the population that lives outside a given time threshold from the hub of resources” [11]. It represents the percentage of population covered within the given subjective time threshold. A coverage radius indicator has been used [22] to address the coverage problem while modelling the EMS in Isfahan and a threshold time of 8 minutes. Only one additional article relates to the probability of survival, but it relates again to threshold response times to accidents [23], not to potential risks at any particular instance.

Concerning the applications, the problem of deployment has been addressed in the literature in recent years due to the importance of designing correctly the rescue service and of staffing properly the resources, using as indicator the time to rescue [12,24] as a proxy of public health. A related problem is that of diversion, which relates to the necessity to relieve congestion at bases or hospitals by requesting ambulances to transport patients to another facility [20]. The indicator that is used is Recommended Safety Time Threshold. In order to relief overcrowding, an indicator based on average patient waiting time for service may be used [25].

## 3. The Traffic-Based Indicators of Public Health

The objective of this article is to keep on defining new indicators that could be used for policy development and for correct staffing of ambulances based on the dynamic system behavior. In particular, this article proposes a set of indicators that are based on the continuous calculation of time lines. Two new indicators to measure the level of service are developed and classified as dynamic population risk indicators, in the sense that the value of the risk (or coverage of the population and/or towns) will not be constant over time. One additional indicator of cost is defined based on the utilization and availability of the ambulances.

### 3.1. Isochrones: Time Lines

**Figure 3:**
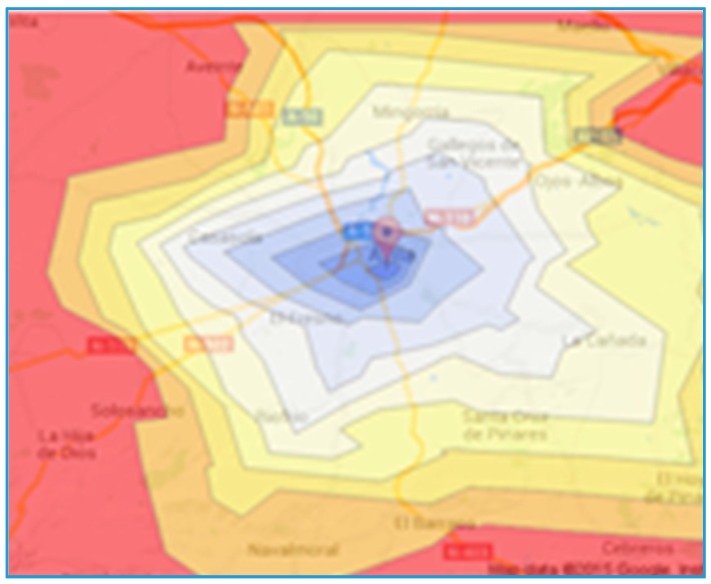
Isochrones over the capital city of Ávila.

The isochrones are the time lines that connect points that are equidistant from a given location. The distance is measured in terms of travel times, not physical distances. If several time thresholds are used, the corresponding graphical display over a map gives an idea of the possibilities of reaching the locations (roads or towns) in time. Figure 3 represents the map of the roads closest to the city of Ávila, where the experimental validation of the indicators is taking place. The isochrones are depicted in increments of 5 minutes. The color coding gives an idea of longer distances. The red color for example represents the area outside 50 minutes.

Therefore one first component of the family of indicators is the isochrones, which might be defined and drawn at regular time intervals. We call this first indicator as ISOCHRONE-K, where K is the value of the time corresponding to the kth time line.

The original idea of this article is that the isochrones are dynamic and are penalized as a function of the road infrastructure and the availability of the ambulances, which is not only related to the probability distribution of occurrence of accidents but also to the probability distribution of the emergency rescue times. Therefore the isochrones must be continuously adapted and calculated, and then the values averaged over time.

In the case of the ambulance emergency system, for each location in the map (roads, bases, hospitals), the travel time is calculated as the time distance from the first available ambulance. Then, those locations that have the same time value are connected with straight lines.

When at least an ambulance is free, the calculation is performed from the location of the closest one, which is usually that at the base, where the ambulances become available after coming back from the hospital. If none is free, the time is calculated from the location where the ambulance becomes available (base or hospital) and a time penalty is added corresponding to the time to reach that point from the location of the closest ambulance. The calculations are performed for each and every ambulance and the minimum time is used. The isochrones might be drawn at a particular moment in time or for a given period. In this last case, the individual values are averaged over time.

### 3.2. Coverage of the Population

Using the traditional static population coverage as the initial measure, the new idea is to incorporate dynamic conditions to its calculation, that is, measure the risk according to the dynamic isochrones. As a result, the proposed dynamic population coverage will then be calculated as the time-weighted average of the instantaneous population coverage.

Given a total population within the region of the map under study, which is the sum of the population of every city or town, the percentage of population covered is that within reach of a certain time threshold or dynamic ISOCHRONE-k, represented by φ_k_:

(1) $$\varphi_{k}=\frac{{\text{PopulationCovered}}_{k}}{\text{Population}}$$

For the case of the EMS, we are going to define six levels in minutes, k = 10, 20, 30, 40, 50, 60. The maximum level corresponds to the “Golden Hour”.

It is worth remarking that the total population is also going to vary over time due to local holidays or sporting events, as well as during weekends; in any of these cases, the floating population that lives elsewhere but have a second residence in the city or town under study or the visiting tourists may raise the population to be covered significantly. 

**Figure 4:**
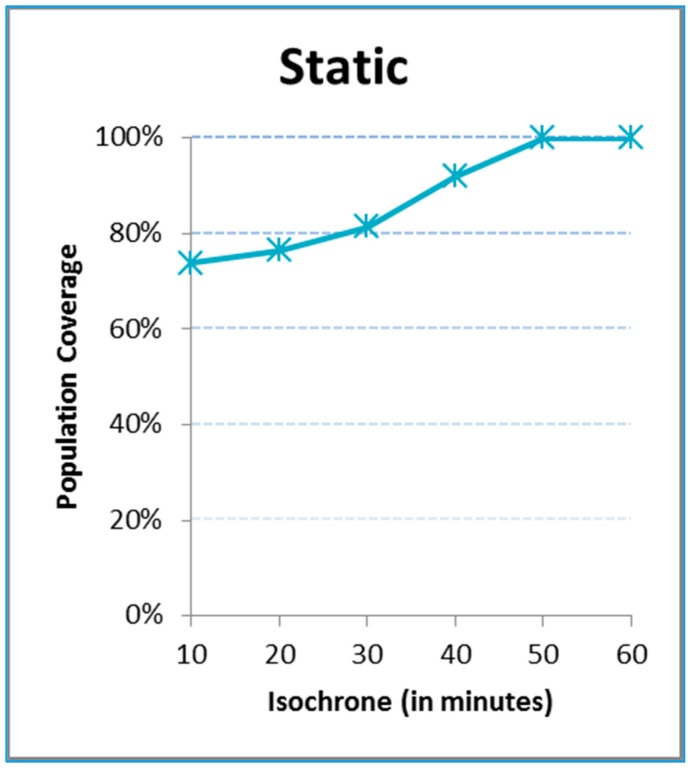
Static coverage of population.

Figure 4 includes the static coverage function for the region of Ávila under analysis, that is, under perfect conditions and with at least one ambulance always available. The cumulative function of the coverage is ascending as a function of the time covered by the isochrone. Of course, the higher the time threshold is, the higher the coverage of the population is. For example, about 75% of the population lives within 10 minutes of the hub and 100% within 50 minutes.

This static graph does not change even if more ambulances are staffed. This indicator however will be penalized with dynamic conditions, shifting the function to the right; to compensate, the number of ambulances should go up. Besides, this indicator does not give a hint on what village, town or city is covered within a given isochrone.

### 3.3. Time Coverage of a Town

Since the population is not evenly distributed over the region under study, it makes sense to define a related indicator that analyses the coverage of just a given city or town. For each town, the whole population is highly concentrated so all the inhabitants are either covered or not at once by an isochrone-k. Accordingly, we define the coverage of a town, π_k_, as the percentage of time (not as a percentage of the population) that a given town is covered by a particular isochrone-k during a particular frame of time:

(2) $$\pi_{k}=\frac{{\text{TimeCovered}}_{k}}{\text{Timeframe}}$$

**Figure 5:**
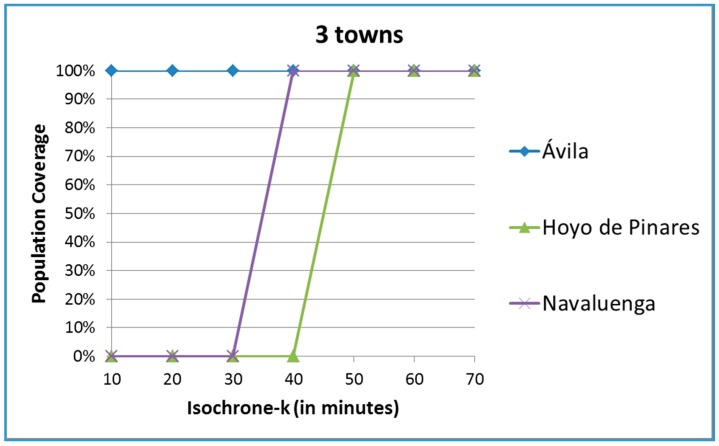
Coverage of three towns of the province of Ávila.

The indicator at a particular moment of time takes a value of 0 if the town is not covered by the isochrone and 1 otherwise. The average indicator over time is what makes sense in this case. Figure 5 includes for illustrative purposes three different towns. The city of Ávila has all of its population within 10 minutes of the hub, so the coverage is 100% for all levels of k under static conditions. Navaluenga lies between 30 (0% coverage) and 40 minutes (100% coverage) away from the hub and Hoyo de Pinares between 40 and 50 minutes away.

The risk of these two towns may increase whenever dynamic conditions are included in the calculations. The static coverage however does not vary even if more ambulances are available of if the population increases.

### 3.4. Occupation of Ambulances

To understand the behavior of the ambulance in terms of availability and have a measure of cost, a new indicator is defined based on the utilization ratio of the ambulance. The following four stages represent the ambulance activities:

1. Travelling time: tasks 2, 4, 6;
2. Preparation time: task 1;
3. Assistance time at the location of the accident and at the hospital: tasks 3 and 5;
4. Free time or available time at which the ambulance is ready to accept an incoming call.

**Figure 6:**
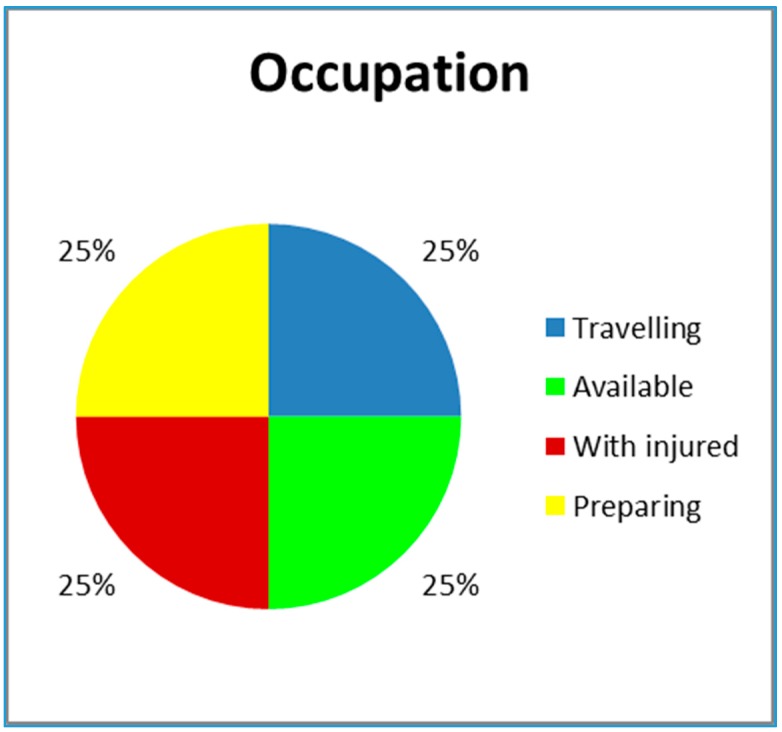
A representative pie-chart of possible states of the ambulance.

A pie-chart will be used to visually represent the four stages. Figure 6 includes an example in which all four stages are evenly distributed. The green color shows availability and therefore percentage of time in which the population is not at risk. Obviously, this slice of the chart increases with the number of ambulances.

## 4. The Simulation Model

In order to study a given EMS under its random environmental conditions via the calculation of the proposed family of indicators, the dynamic time-based conditions are better represented within a discrete-event simulation environment [12,15,16]. In this case, we have chosen a multipurpose programming language like C++ to develop the model, with input and output tables in MS Excel. First, the way the map has been implemented is shown. Then, the randomness related to the movement and availability of the ambulances is explained and depicted using web technology. All the necessary data is primarily obtained from open access sources.

### 4.1. The Infrastructure of the EMS

The abstraction process of the EMS starts with the definition of the map that represents the region that is going to be analyzed with its roads. The map is easily represented with a grid composed of small cells that cover the whole region. Each individual cell might hold:

1. The hub
2. The cities or towns
3. The roads
4. An ambulance
5. An accident
6. A hospital

### 4.2. The Movement of the Ambulance

The key to the model is the movement of the ambulance between the hub, the accident sites and the hospitals, as well as its location along the way. In other words, the ambulance moves between cells on the map. To calculate the travel times, a second parallel grid of cells represents the time distances to the hub. The values in each cell include not only the quickest time but added penalties incurred due to imperfect traffic conditions. The travel times between two points are then readily calculated.

It is worth mentioning that the way this matrix of time distances is defined and modelled is based on the underlying assumption that the resources are intelligent and always select the shortest route in terms of the response time. Then this shortest route is followed and at each of the cells of the path a time delay is assigned corresponding to the travel time of the ambulance along the road.

Besides the travel times, the ambulance might be held for an interval of time [26] when actuating at the corresponding cell:

1. At the hub for preparation: represented by a lognormal distribution; lognormal (2.5, 1).
2. At the accident site: time represented by a uniform distribution; uniform (23.2, 37.2).
3. At the hospital: time that is represented by a triangular distribution; triangular (12.7, 13.7, 21.9).

### 4.3. Generation of Accidents

The movement of an ambulance starts with the generation of an accident. A third matrix of values includes the accident rate at each of the road cells. The probabilistic occurrence of accidents is usually considered to be best represented using a Poisson random variable, Po (λ) [27]. Its parameter λrepresents and is calculated as the average number of events within a given period of time.

There exist official statistics that summarize the historical accidents for each and every road per period of time. If the numbers are aggregated in such a way that the level of detail is not enough, there is a need to ask the authorities for further detail.

### 4.4. The Population

Static population data for each of the towns and their associated cells should be obtained from the official local databases and may be validated in the individual town webpages. Additional sources of data are needed in order to estimate variability over time. We tried at first to use electricity consumption but we could not find available data at the local level.

We have selected instead the official data about hotel stays, which is included usually in the national statistics body or even at the European level [28]. The monthly hotel stays clearly relate to journeys during holiday periods, increasing the probability of accidents. We have assigned all the stays to the weekends and local holidays.

Besides, we have also included the sporting events as a source of a population increase. On top of that, the population figures during weekends and holidays have been increased by a 20% to account for floating population.

### 4.5. Meteorology

The historical values on how many days within a month an event has occurred are available in public databases. The values that are used to represent the adverse meteorological events are included in Table 1. There is a duration that represents how long the event lasts and a penalty factor that is used to multiply the time matrix at the corresponding cells where the event takes place. For example, if it snows, the static travel times are tripled.

**Table 1:** Meteorological events.

Event	Static	Rain	Snow	Fog	Ice
Duration (minutes)	720	120	240	120	240
Penalty factor	1	2	3	3	4

With the count of days per month and the durations of the events, the rate of occurrence of the different events may be modelled with a Poisson distribution and the time between snows with an exponential distribution. A Monte Carlo generator might then be used to determine both the start time of an event and its corresponding duration.

### 4.6. Congestion and Festivities

When an accident occurs, the times to travel through the cell where it takes place, as well as through the surrounding cells are doubled to account for congestions. The travel times are also doubled whenever there are sporting or religious activities that partially block certain roads.

### 4.7. The Flowchart of the Model

The simulation model is event driven and programmed in C++. There exists a list of future events and the model jumps to the next executable event. Each event is represented with a module, which abstracts certain logic and generates additional events in the list. The modules are:

1. Data updating: monthly, the data about each source of randomness is updated; however certain time penalties due to congestions are updated daily.
2. Accident generation: an accident is provoked penalizing the time to drive through the roads affected by the accident due to congestion, and calls an ambulance. The assignment of accidents to ambulances is on a FIFO basis. The first accident that takes place is the first one that is going to be attended. Besides, the ambulance that is assigned is the one that is closest. In our case, it is always the case that the ambulance is at the base, since we free it when it is back at the hub from the hospital. The required number of ambulances are assigned to an accident, and they go from the site of the accident to the closest hospital.
3. Meteorology generation: generates events and how long they last.
4. Ambulance rescue activities: it covers the six stages related to the movement of the ambulance while giving service to an accident.
5. Indicators collection: at each event, the indicators are updated for each cell individually and for the map as a whole.
6. Results visualization: at the end of the simulation, the aggregate indicators are calculated and exported for proper visualization.

## 5. Experiment in Ávila, Spain

Ávila is a province in Spain located about 100 kilometers northwest of Madrid. Figure 1a includes the road map around the capital and main city, Ávila, where the ambulance base is located, as well as the main population nuclei. The calculation of the set of indicators is going to cover 2014 and the main sources of data are going to change mainly on a monthly basis, although some values about festivities are updated daily.

### 5.1. Static Conditions

**Figure 7:**
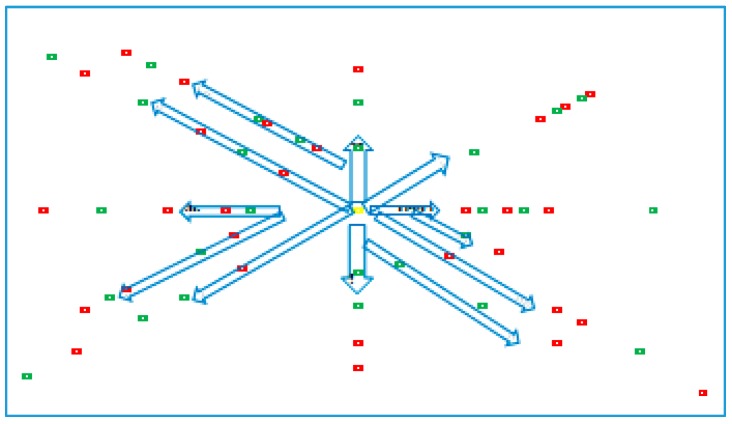
Selected points for the simulation.

The first step is to convert the Google map into the input matrix of cells using the GPS coordinates (Figure 7). Several locations are selected to generate accidents: the base is represented in yellow, the towns in red and the road points in green. The hospital is located by the base.

**Figure 8:**
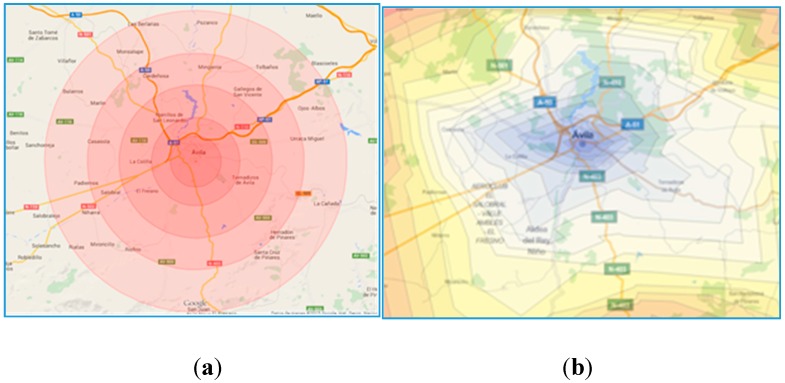
(**a**) Radial isochrones; (**b**) Static isochrones.

The travel times from the hub associated to each of the locations are obtained using Google Maps. These time distances are then drawn as radial isochrones (Figure 8a) or as static isochrones (Figure 8b). The radial isochrones are drawn using the average distance to the points in the roads.

### 5.2. Dynamic Conditions

The first source of variability is that of accidents. We could not collect data from the official web for 2014, but got it from the local authorities [29]. The accidents are summarized in Table 2 for proper input into the simulation.

**Table 2:** Accidents in 2014.

Roads	JAN	FEB	MAR	APR	MAY	JUN	JUL	AUG	SEP	OCT	NOV	DEC
**TOTAL**	**53**	**44**	**65**	**56**	**72**	**66**	**61**	**58**	**66**	**76**	**54**	**74**
A-50	3	3	3	2	6	1	4	2	3	0	1	3
A-51	0	2	0	2	1	0	2	1	0	2	1	1
A-6	2	4	2	3	3	3	3	4	8	2	2	3
AP-51	0	0	0	0	0	1	0	0	0	1	0	2
AP-6	3	0	2	2	0	1	1	1	2	1	1	1
AV-110	1	2	4	0	0	0	1	0	3	2	1	2
AV-500	1	0	0	0	1	1	1	2	2	1	3	2
AV-502	0	4	5	2	0	1	3	7	1	1	1	0
AV-503	1	0	0	0	0	0	0	0	1	1	2	4
AV-804	1	0	1	3	2	0	1	2	4	0	3	1
AV-900	4	1	1	0	0	0	3	2	2	2	1	2
AV-902	1	1	1	4	0	0	2	3	0	1	1	0
CL-501	8	7	4	7	8	4	6	10	4	4	15	9
CL-505	2	4	4	2	1	0	4	3	4	2	6	1
N-110	15	10	11	10	7	9	11	8	9	7	9	7
N-403	8	7	5	5	4	4	5	5	7	13	6	5
N-501	0	1	0	0	1	0	3	1	0	1	1	3
N-502	10	8	2	1	5	6	5	5	5	5	2	8

The static population data for the towns is obtained from the official database [30] and validated via the individual town webpages. The variations of population are calculated using the official data about hotel stays [31]. The population percentages are the values that will be used to update each town individually. Table 3 includes the corresponding calculation for the six largest towns of the province, which account for 90% of the total population covered within the EMS, and all are above 1000 inhabitants. Avila officially has 58,933 inhabitants for a 74%, but in its calculation, most of the 79,913 hotel stays are also assigned to the capital (the assignment is proportional to the population). In January for example, Ávila holds some 59,000 inhabitants plus 74% of the 18,000 hotel stays for a rounded total of 73,000.

**Table 3:** Population in 2014 (in thousands).

Towns	Pop	% abs	% cum	JAN	FEB	MAR	APR	MAY	JUN	JUL	AUG	SEP	OCT	NOV	DEC
Ávila	59	74	74	73	76	87	92	90	86	93	104	94	93	82	82
El Tiemblo	4	5	79	5	6	6	7	7	6	7	8	7	7	6	6
Hoyo de Pinares	2	3	82	3	3	3	4	4	3	4	4	4	4	3	3
Navaluenga	2	3	85	2	3	3	3	3	3	3	4	3	3	3	3
Piedrahita	2	2	87	2	3	3	3	3	3	3	4	3	3	3	3
El Barraco	2	2	90	2	3	3	3	3	3	3	4	3	3	3	3
Hotel Stays (province)				18	24	38	45	42	37	46	61	48	46	31	32

The overall population figures are further adjusted to account for local festivities and other sporting or religious events (Table 4). The numbers are aggregated for the whole region for ease of representation. Besides, weekends are also included in order to better represent floating population.

**Table 4:** Local festivities and events in 2014 (in days).

Type	JAN	FEB	MAR	APR	MAY	JUN	JUL	AUG	SEP	OCT	NOV	DEC
**TOTAL**	0	6	12	12	20	41	14	18	39	17	0	0
Local events	0	4	0	1	10	12	1	6	19	11	0	0
Roads closed	0	2	12	11	10	29	13	12	20	6	0	0

The number of days per month of the different meteorological events has been obtained from the corresponding authority through its webpage (Table 5) [32].

**Table 5:** Meteorology in 2014.

Days/Month	JAN	FEB	MAR	APR	MAY	JUN	JUL	AUG	SEP	OCT	NOV	DEC
Rain	5.9	5.0	4.2	7.8	9.0	4.7	1.9	2.6	4.4	7.6	7.1	6.7
Snow	4.5	4.3	2.3	2.2	0.4	0.0	0.0	0.0	0.0	0.1	1.9	3.2
Storms	4.5	2.3	1.5	1.2	1.0	0.6	0.3	0.3	0.7	1.3	3.4	4.1
Fog	20.1	16.4	12.6	6.6	1.7	0.1	0.0	0.0	0.3	1.7	11.5	16.3

## 6. Results and Discussion

The simulation model is run for one year, 20 times to methodologically account for variability [33]. In this section we show results to demonstrate the applicability of each of the indicators and to study the deployment and staffing of the ambulances as a test case.

### 6.1. No Accidents

**Figure 9:**
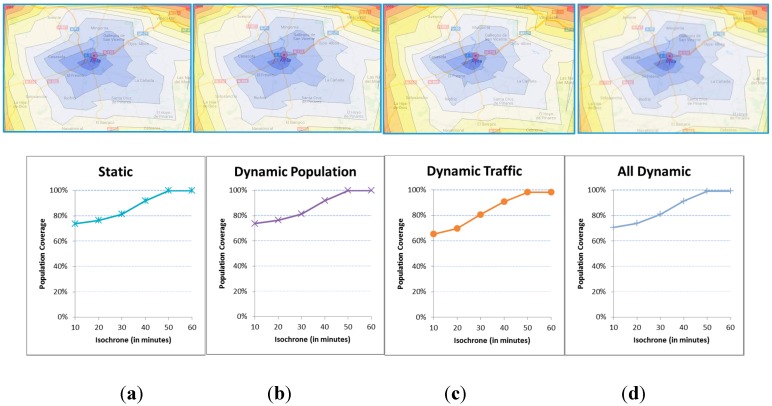
(**a**) Static population and standard times; (**b**) Dynamic population and standard times; (**c**) Static population and dynamic times; (**d**) Dynamic population and dynamic times.

Figure 9 includes the isochrones that result from different executions of the model, all of which do not include accidents. We vary and combine the sources of randomness due to variations in the population and the travel times or traffic conditions. The differences are not important but it is true that the functions cover somewhat less population when the variability is applied.

### 6.2. Deployment

**Figure 10:**
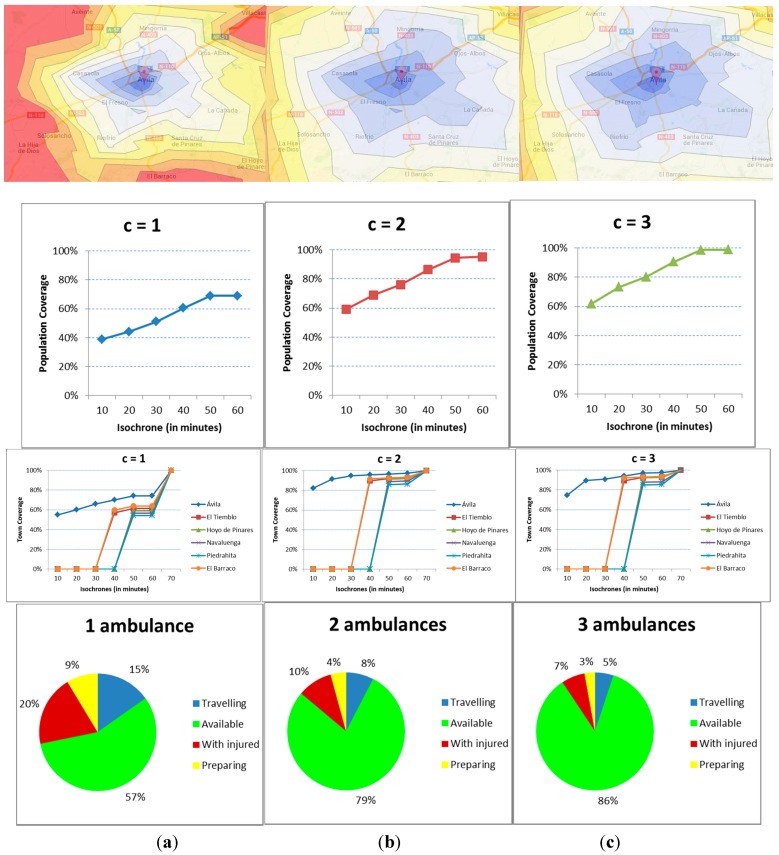
(**a**) Results for one ambulance; (**b**) Results for two ambulances; (**c**) Results for three ambulances.

This section shows the analysis of the EMS as a function of the number of ambulances *c* that may be staffed at the hub, either c = 1, c = 2 or c = 3. Figure 10 includes the results for all four indicators, with each column including the results for each staffing level. In the first row of graphs, the isochrones that result from different executions of the model are shown, with the population coverage included in the second, the town coverage in the third and the utilization ratios in the fourth.

The results show a vast improvement with a second emergency unit. With only one, and even if the rate of accidents is low, the ambulance is unavailable for a considerable amount of time, reducing the coverage and consequently increasing the health risk. The addition of a third ambulance however does not improve the situation enough to be considered economically appropriate.

The isochrones show several areas that, on average terms, the ambulances cannot reach within 50 minutes. That is not the case with more ambulances. Sure enough, the population covered within 20 minutes is just 45% with c = 1 but above 70% for c = 2 and c = 3. The Golden Hour only covers 70% with one ambulance, 96% with two and almost 100% with three.

The town coverage gives additional insights to the problem. The indicator for the main city vastly improves, with the coverage rising from 60% to 90% and 96% for isochrone-20. For the rest of the towns the improvement is for isochrone-40 with five villages being above the 90% coverage, and all of them above that mark for isochrone-50.

In terms of availability of the ambulances, the value is 57%, 79% and 86%, respectively, for the different resource levels. As for the busy times, the time at the site of the accident is greater than the travelling time, even if the latter is penalized. Therefore, it looks like the availability is the key to correctly staff the ambulances.

**Figure 11:**
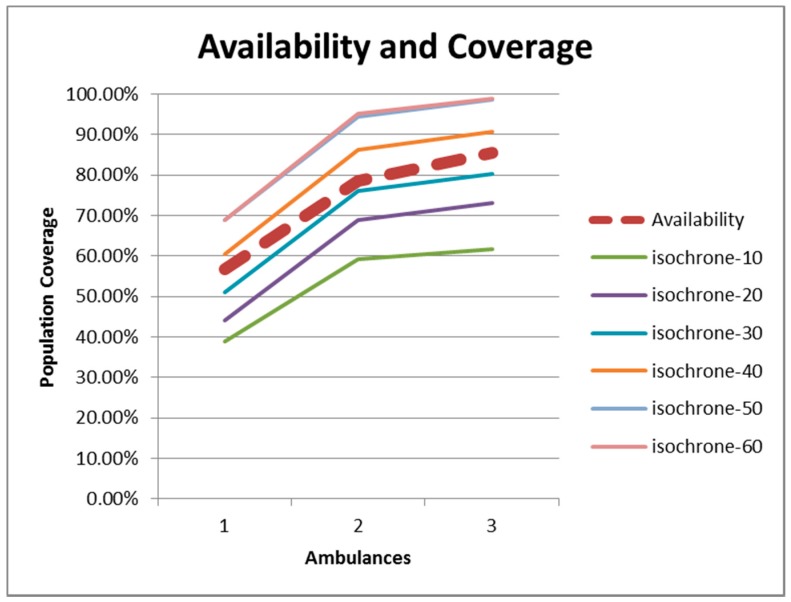
Relationship between population coverage and percent availability.

As a summary, Figure 11 includes the relationship between availability (dashed line) and population coverage. The patterns of the functions are the same. For this region in Spain, there is a relationship that can be set just above the isochrone-30 level. In other words, if the isochrone-30 is the threshold, the population coverage and the availability are both around 55% for c = 1, 75% for c = 2 and 85% for c = 3.

Remember however that the Spanish legislation aims at covering at least 85% of the population within 20 minutes. The size of the region and the road infrastructure make it in this case complicated and very costly to raise the 85% coverage level from isochrone-30 to isochrone-20.

### 6.3. Sensitivity Analysis

**Figure 12:**
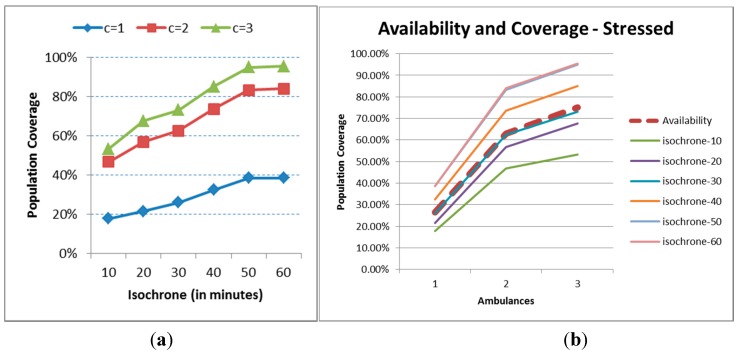
Population coverage for the stress scenario (**a**) Population coverage; (**b**) Availability.

A further stress analysis is made in order to assess the robustness of staffing two ambulances. All the time figures (task times and penalties due to events) are multiplied by two. Accordingly, the dynamic indicators for public health will all be somewhat worse. The stress effect on the results for just one ambulance (Figure 12a) is very important, with the indicator never reaching the 40% coverage. The differences between c = 2 and c = 3 are just about 10% for isochrones greater than or equal to 20 minutes, and are generated basically by travel times and not the meteorological events. It does not however look like the cost of implementing a third ambulance is mandatory. In terms of availability, it coincides in this case almost perfectly with the 30 minutes isochrone.

## 7. Conclusions

Negative situations relative to public health are frequently mentioned in local newspapers. For example, a three year-old baby lost its life due to the lack of availability of an ambulance for 20 minutes in the small town of Elche in the South of Spain on July 28th, 2015. The time to rescue is considered unacceptable and the authorities ask for the deployment of “all the medical means that are necessary… to minimize the risky situations… since nowadays the resources are not proportional to the population” [34]. At the moment of the happenings, the only unit was occupied and the requested permanent additional ambulance had been deployed to a close town to cover for the increased population by the beach during the summer months.

The original idea of this ongoing research has been therefore to include both the availability of the ambulances, the dynamic changes in population as well as the randomness in travel times due to weather or congestions in the calculation of a new set of indicators for the assessment of the influence of traffic accidents in public health. We have defined and developed a family of indicators based on dynamic isochrones, which are also very appealing in graphical format. The coverage of the population as a whole can be calculated, but also a different indicator is used for small town based on coverage time. The occupation of ambulances, as an indicator of cost, is also incorporated in the analysis.

After applying the framework to a small region in Spain via a simulation model, it appears that the key indicator is that of the percent availability of the ambulances, since the population or towns are at risk whenever all the ambulances are busy while in an emergency rescue. We are currently improving the developed model with an additional aim to apply it to other towns in Spain with different infrastructures and populations to compare the validity of results, and hopefully establish a relationship between the level of deployment and the characteristics of the area that needs to be covered

As a continuation, we are also changing the model so it can be used for other EMS decision making scenarios, like the assignment decisions in the short-term, for example, the relocation of ambulances, or even while controlling the position and the reach of the emergency units online.
